# A critical time window for the analgesic effect of central histamine in the partial sciatic ligation model of neuropathic pain

**DOI:** 10.1186/s12974-016-0637-0

**Published:** 2016-06-24

**Authors:** Jie Yu, Ying-Ying Tang, Ran-Ran Wang, Guo-Dong Lou, Ting-Ting Hu, Wei-Wei Hou, Jia-Xing Yue, Hiroshi Ohtsu, Li-Yun Shi, Shi-Hong Zhang, Zhong Chen

**Affiliations:** Department of Pharmacology, Key Laboratory of Medical Neurobiology of the Ministry of Health of China, School of Basic Medical Sciences, Zhejiang University, Hangzhou, 310058 Zhejiang China; College of Basic Medical Science, Zhejiang Chinese Medical University, Hangzhou, 310053 Zhejiang China; Collaborative Innovation Center for Diagnosis and Treatment of Infectious Diseases, First Affiliated Hospital, School of Medicine, Zhejiang University, Hangzhou, China; Department of Engineering, School of Medicine, Tohoku University, Aoba-ku, Sendai, 980-8775 Japan; Department of Basic Medical Science, Hangzhou Normal University, Hangzhou, 311121 Zhejiang China

**Keywords:** Analgesic effect, Central histamine, Histidine, Neuropathic pain, Microglial activation, IL-1β

## Abstract

**Background:**

It is known that histamine participates in pain modulation. However, the effect of central histamine on neuropathic pain is not fully understood. Here, we report a critical time window for the analgesic effect of central histamine in the partial sciatic nerve ligation model of neuropathic pain.

**Methods:**

Neuropathic pain was induced by partial sciatic nerve ligation (PSL) in rats, wild-type (C57BL/6J) mice and HDC^−/−^ (histidine decarboxylase gene knockout) and IL-1R^−/−^ (interleukin-1 receptor gene knockout) mice. Histidine, a precursor of histamine that can increase the central histamine levels, was administered intraperitoneally (i.p.). Histidine decarboxylase (HDC) enzyme inhibitor α-fluoromethylhistidine was administered intracerebroventricularly (i.c.v.). Histamine H_1_ receptor antagonist mepyramine and H_2_ receptor antagonist cimetidine were given intrathecally (i.t.) and intracisternally (i.c.). Withdrawal thresholds to tactile and heat stimuli were measured with a set of von Frey hairs and infrared laser, respectively. Immunohistochemistry and Western blot were carried out to evaluate the morphology of microglia and IL-1β production, respectively.

**Results:**

Histidine (100 mg/kg, i.p.) administered throughout days 0–3, 0–7, or 0–14 postoperatively (PO) alleviated mechanical allodynia and thermal hyperalgesia in the hindpaw following PSL in rats. Intrathecal histamine reversed PSL-induced thermal hyperalgesia in a dose-dependent manner and intracisternal histamine alleviated both mechanical allodynia and thermal hyperalgesia. Moreover, α-fluoromethylhistidine (i.c.v.) abrogated the analgesic effect of histidine. However, histidine treatment initiated later than the first postoperative day (treatment periods included days 2–3, 4–7, and 8–14 PO) did not show an analgesic effect. In addition, histidine treatment initiated immediately, but not 3 days after PSL, inhibited microglial activation and IL-1β upregulation in the lumbar spinal cord, in parallel with its effects on behavioral hypersensitivity. Moreover, the inhibitory effects on pain hypersensitivity and spinal microglial activation were absent in HDC^−/−^ mice and IL-1R^−/−^ mice. H_1_ receptor antagonist mepyramine (200 ng/rat i.t. or i.c.), but not H_2_ receptor antagonist cimetidine (200, 500 ng/rat i.t. or 500 ng/rat i.c.), blocked the effects of histidine on pain behavior and spinal microglia.

**Conclusions:**

These results demonstrate that central histamine is analgesic within a critical time window in the PSL model of neuropathic pain via histamine H_1_ receptors. This effect may partly relate to the inhibition of microglial activation and IL-1β production in the spinal cord following nerve injury.

**Electronic supplementary material:**

The online version of this article (doi:10.1186/s12974-016-0637-0) contains supplementary material, which is available to authorized users.

## Background

Neuropathic pain is a significant clinical problem. Typical manifestations of neuropathic pain, such as thermal hyperalgesia, mechanical allodynia, and unbearable burning pain, are lead symptoms of complex regional pain syndrome 2 (CRPS-2) [1–3]. Approximately 15 % of sufferers have unrelenting pain, and overall 30 % of patients who worked before CRPS-2 onset remain completely unable to work [4]. Since the pathophysiology of neuropathic pain in CRPS-2 is still poorly understood, clinical treatment is largely limited. Intriguingly, however, physical therapy or medication treatment is clinically much more effective if started soon after the onset of symptoms, indicating the significance of early pathophysiological processes and early intervention for the neuropathic pain of CRPS-2 [5, 6].

Central histaminergic neurons are located in the tuberomammillary nucleus of the posterior basal hypothalamus, and their fibers project widely to different brain regions and the spinal cord [7]. Previous studies have uncovered that histamine may participate in pain modulation. It was reported that a higher dose of histidine, which is the precursor of histamine and can increase histamine levels in the central nervous system (CNS) [8–10], but not a lower dose, suppresses both phases of the pain responses in the formalin test [11]. Malmberg-Aiello et al. also reported an antinociceptive effect of peripherally loaded histidine in paw pressure, abdominal constriction, and hot plate tests in rats and mice [12]. Moreover, brain histamine was found to be analgesic in a rat model of acute trigeminal pain [13]. As for neuropathic pain, Huang et al. found that, 2 weeks after nerve injury, a low dose of histamine given intracerebroventricularly (i.c.v.) decreases while a high dose of histamine increases the nociceptive threshold to mechanical stimulation [14]. These findings reflect the acute or transient effect of central histamine on pain sensation in the context of neuropathic pain, but its effect on the development of neuropathic pain remains unclear. Recently, we reported that central histamine is analgesic in a rat model of phantom pain, suggesting a potential role of central histamine in the development of spontaneous neuropathic pain [15]. We hypothesize that central histamine might be a member among a variety of factors [16, 17] that are involved in pathophysiological processes in the CNS following nerve injury and contribute to the development of neuropathic pain in CRPS-2.

In laboratory studies, models of tibia fracture/cast or chronic post-ischemia pain have been adopted to recapitulate the vascular, trophic, inflammatory, and painful aspects of CRPS [18, 19]. Based on the association of CRPS-2 with nerve injury, a partial sciatic nerve ligation (PSL) model, which has been widely used to induce experimental neuropathic pain since established by Seltzer et al. in 1990, has been adopted as an animal model to mimic the neuropathic pain component of CRPS-2 [20–22]. Therefore, the present study was designed to investigate the effect of central histamine on pain hypersensitivity in the hindpaw induced by PSL, looking forward to shedding more light on the pathogenesis and treatment of neuropathic pain in CRPS-2.

## Methods

### Animals

Adult Sprague-Dawley rats (260–300 g, grade II, Certificate No. SCXK2003-0001, Experimental Animal Center, Zhejiang Academy of Medical Science, Hangzhou, China), histidine decarboxylase knockout (HDC^−/−^) and IL-1 receptor knockout (IL-1R^−/−^) mice and their wild-type littermates (C57BL/6J), all male and aged 8–12 weeks, were used in this study. The rats and mice were kept under a 12-h light-dark cycle (lights on from 08:00 to 20:00). Water and chow were given ad libitum. All experiments were in accordance with the Guide for the Care and Use of Laboratory Animals of the National Academy of Sciences (National Research Council, 1996) and were approved by the Animal Care and Use Committee of Zhejiang University. Efforts were made to minimize the number of animals used and their suffering.

### Surgery

Under deep anesthesia with inhalation of isoflurane and aseptic conditions, the left sciatic nerve was exposed at high-thigh level and partially ligated as previously described [20]. Briefly, the dorsum of the nerve was carefully freed from surrounding connective tissues at a site near the trochanter, just distal to the point at which the posterior biceps semitendinosus (“PBST”) nerve branches off the common sciatic nerve. Using honed (no. 5) jewelers’ forceps, the nerve was fixed in its place by pinching the epineurium on its dorsal aspect. A 6-0 silicon-treated silk suture (8-0 silk suture for mice) was inserted into the nerve with a 3/8 curved, reversed-cutting mini-needle, and tightly ligated so that the dorsal 1/3–1/2 of the nerve thickness was trapped in the ligature. The sciatic nerve in the sham group was exposed but left intact. The wound was closed in layers. The animals were then placed back to their individual cages after recovered from anesthesia in a warm incubator.

### Assessment of PSL-induced pain-like behaviors

Behavioral tests (*n* = 7–9 animals/group) were carried out 2–3 h after daily administration of histidine between 11:00 and 17:00, 3 days preoperatively and on days 1, 3, 7, and 14 postoperatively (PO) by blinded examiners. Animals were placed in a chamber with a mesh metal floor (20 × 30 cm for the rats and 8 × 8 cm for the mice), covered by an opaque plastic dome 10-cm high, and were allowed to habituate for 1 h before tests.

Withdrawal threshold to tactile stimulation was measured with a set of von Frey hairs with a bending force ranging from 2.0 to 26.0 g for the rats and from 0.008 to 1.4 g for the mice. Stimulation was applied to the plantar surface of the ipsilateral hindpaw. Each hair was indented in the mid-plantar skin until it just bent. Clear paw withdrawal, shaking, or licking was considered as a nociception-like response. According to Wang et al. [23], the filament of 8 g was used first for the rats and 0.16 g for the mice. The stimulation was applied five times (several seconds for each trial) with an interval of at least 5 min. The strength of the next filament was decreased if the animal responded or increased if the animal did not respond. The minimum strength that evoked nociceptive responses at least three times out of the five trials was considered as the mechanical withdrawal threshold. Animals that did not respond to all filaments were given a maximal strength of 26 g for the rats and 1.4 g for the mice.

Withdrawal threshold to heat stimulation was determined by beaming a single short pulse of infrared laser (1.5 mm in diameter, pulse width 200 ms for the rats and 150 ms for the mice, 14–31 A; LPYE, China) to the plantar surface of the ipsilateral hindpaw [24]. The laser was guided by a visible aiming beam, which illuminated the target with a red spot. The stimulation was applied three times with an interval of at least 5 min. The threshold was determined by increasing the current intensity (*A*) by a step until the withdrawal response was induced at least two times out of the three trials of stimulation. The averaged threshold from these three trials was recorded as the thermal nociception threshold.

### Determination of histamine concentration in the central nervous system by high-performance liquid chromatography

After treated with histidine for 3 days, rats were perfused intracardially with PBS (pH 7.4) under anesthesia by chloral hydrate (400 mg/kg, intraperitoneally (i.p.)). The spinal cord at L4–L5 and medulla segments was rapidly removed and stored at −80 °C until assay. Tissues were homogenized with 0.4 mol/l perchloric solution and centrifuged at 12,000*g* for 20 min at 4 °C, and the supernatant was collected. Analysis of histamine was performed by high-performance liquid chromatography (HPLC) as described previously [25]. The HPLC was controlled, the concentration below the minimum detectable level was given a concentration of 10 ng/g, and the data were acquired and analyzed using Coul Array software (ESA, Chelmsford, MA, USA). All equipment was obtained from ESA (Chelmsford, MA, USA).

### Immunohistochemistry

Under anesthesia by chloral hydrate, rats and mice were perfused intracardially with PBS (pH 7.4) followed by 4 % paraformaldehyde. The spinal cord at L4/L5 segment was dissected and post-fixed in the same fixative overnight at 4 °C, then cryoprotected by infiltration with 30 % sucrose overnight. Cryostat sections were cut at 16 μm and incubated with 3 % normal donkey serum (dissolved in PBS) containing 0.3 % Triton X-100 for 2 h, then incubated with rabbit anti-Iba-1 IgG (Wako; 1:1000) overnight at 4 °C and anti-rabbit IgG-Alexa Fluor 488 (Invitrogen; 1:400) for 2 h at room temperature. Finally, Iba-1 immunostaining was observed by a fluorescence microscope (Olympus BX51; Olympus, Tokyo, Japan), and images were captured under identical illumination and exposure conditions. The fluorescence intensity of Iba1 immunoreactivity specifically in the superficial lamina (I–II) of the dorsal horn from L4 and L5 segments was measured using Image J software (v1.37, NIH, USA). The intensity for each section was obtained by averaging the intensity of five fields that were randomly selected. The intensity from two to three slides (three to four sections per slide) was averaged for each animal and then normalized by that of the sham group.

### Western blot analysis

Under chloral hydrate anesthesia, rats were perfused intracardially with PBS. The spinal cord at L4/L5 segment was rapidly removed and then homogenized and lysed in homogenization buffer. Protein concentrations were determined by bicinchoninic acid (BCA) protein assay. After concentration determination and denaturization, protein samples were separated by SDS-PAGE gels and then transferred onto a nitrocellulose membrane. The membranes were incubated with rabbit anti-IL-1β (1:200; Abcam) and glyceraldehyde-3-phosphate dehydrogenase (GAPDH) (1:3000; Kang Chen) overnight at 4 °C and with secondary antibody against rabbit (IRDye 800-coupled, 1:10,000) for 2 h at room temperature. Blots were visualized with Odyssey infrared imaging system (LI-COR Biosciences) and analyzed with the Odyssey software. The ratio between IL-1β and GAPDH was calculated and then normalized to the values measured in the control group.

### Drug administration

Rats were weighed and received histidine, the precursor of histamine, or vehicle postoperatively once daily via intraperitoneal injection in various regimens: (1) started immediately after the surgery and one more injection on the next day (0–1 day), or three more injections on the next 3 days (0–3 days), or till day 7 PO (0–7 days), or till day 14 PO (0–14) and (2) started from 2 days after surgery and one more injection on the next day (2–3 days), or from day 4 PO and lasting 4 days (4–7 days), or from day 8 PO and lasting 7 days (8–14 days). Mice were given histidine intraperitoneally once daily at a dose of 200 mg/kg during the period of 0–7 days PO.

For intracerebroventricular microinjection (i.c.v.) in rats, a stainless steel cannula (Reward, China) was implanted into the left lateral cerebral ventricle (AP −0.96 mm, L −2.0 mm, V −4.0 mm) and then embedded in the skull with dental cement 7 days before nerve injury. For intracisternal microinjection, a small burr hole (1~2 mm in diameter) was drilled in the occipital bone, exposing the meninges. A small stainless steel cannula (Reward, China) was carefully implanted (in a caudal direction) along the internal surface of the occipital bone into the cisterna magna (depth of 7 mm) [26]. Two hours before histidine administration, α-fluoromethylhistidine (α-FMH, kindly provided by Professor C. Kamei, Okayama University, Japan), 50 μg in 10 μL, was injected once daily in 10 min through days 0–7 PO via a disposable dental needle (30 G, Nipro Medical Industries Ltd, Japan), which was attached to a 15–20-cm PE-10 tube fitted to a 25-μL Hamilton syringe. For intrathecal injection (i.t.), rats were briefly anesthetized with isoflurane inhalation, the dorsal fur was shaved, and the spinal column was arched. A 30-gauge needle connected to a 10-μL Hamilton syringe was inserted into the subarachnoid space between the L5 and L6 vertebrae. Needle penetration into the right place was indicated by a tail flick. Histamine (50, 100, 200 ng), mepyramine (100, 200 ng), cimetidine (200, 500 ng), or IL-1β (10 ng), all in 5 μL, was once daily injected in 10 min through days 0–7 or days 0–3 PO. The dosages were determined by pilot studies.

### Statistical analysis

Data are presented as mean ± SEM. Statistical analysis was performed by SPSS for Windows (v 20.0). Statistical significance was determined as follows: (1) two-way ANOVA was used for the comparison of withdrawal thresholds at different time points and one-way ANOVA followed by Dunnet’s test for the comparison of the data between groups (histidine-treated vs saline-treated groups) at the same time points; (2) unpaired *t* test was used for the comparison of Iba-1 fluorescence intensity, IL-1β expression, and concentration of histamine in the medullar and lumbar cord (histidine-treated vs saline-treated groups). The criterion for statistical significance was set at *P* < 0.05.

## Results

### Histidine alleviated nerve injury-induced mechanical and thermal hypersensitivity

**Fig. 1:**
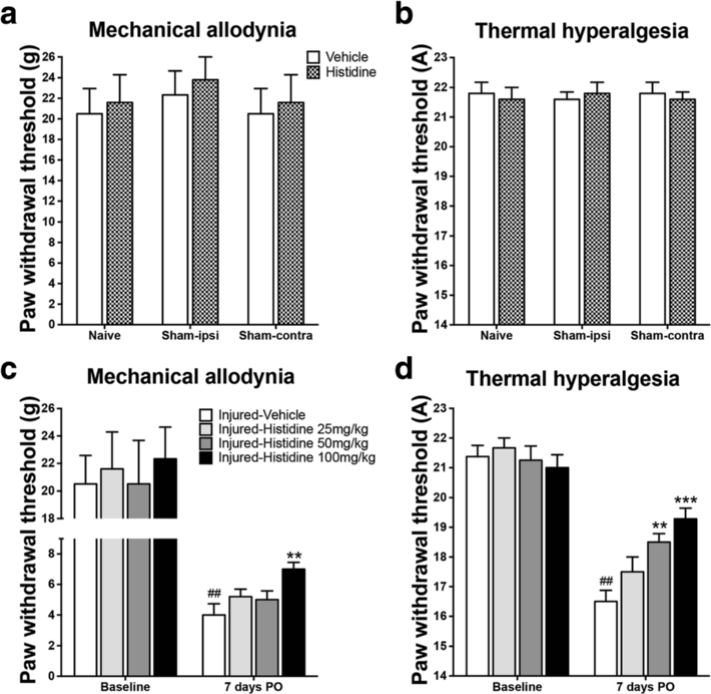
Effects of histidine on thresholds to mechanical and thermal stimuli in rats. Histidine (100 mg/kg, i.p.) administered once daily for three consecutive days did not affect hindpaw withdrawal thresholds to mechanical and thermal stimuli in naïve and sham-operated rats (**a**, **b**). Histidine treatment for 7 days (0–7 days PO) at various doses (25, 50, or 100 mg/kg, i.p.) alleviated mechanical allodynia (**c**) and thermal hyperalgesia (**d**) following PSL. **P < 0.01 and ***P < 0.001, compared with the vehicle-treated group. ^##^ *P* < 0.01, compared with the baseline. *n* = 7–9/group

Consecutive daily administration of histidine (100 mg/kg, i.p.) for 3 days did not affect the paw withdrawal thresholds in response to mechanical and heat stimuli in the sham-operated group (Fig. 1a, b). On day 7 PO, the rats receiving saline (once daily for 7 days) showed a robust decrease in withdrawal thresholds to both mechanical (Fig. 1c) and heat stimuli (Fig. 1d) in the ipsilateral hindpaw, indicating the development of mechanical allodynia and heat hyperalgesia. Histidine, once daily for 7 days, did not show any analgesic effect at the dose of 25 mg/kg (Fig. 1c, d), while alleviated thermal hyperalgesia at the dose of 50 mg/kg. By contrast, 100 mg/kg histidine significantly inhibited the decrease of withdrawal thresholds to mechanical and heat stimuli (*P* < 0.01; Fig. 1c, *P* < 0.001; Fig. 1d).

**Fig. 2:**
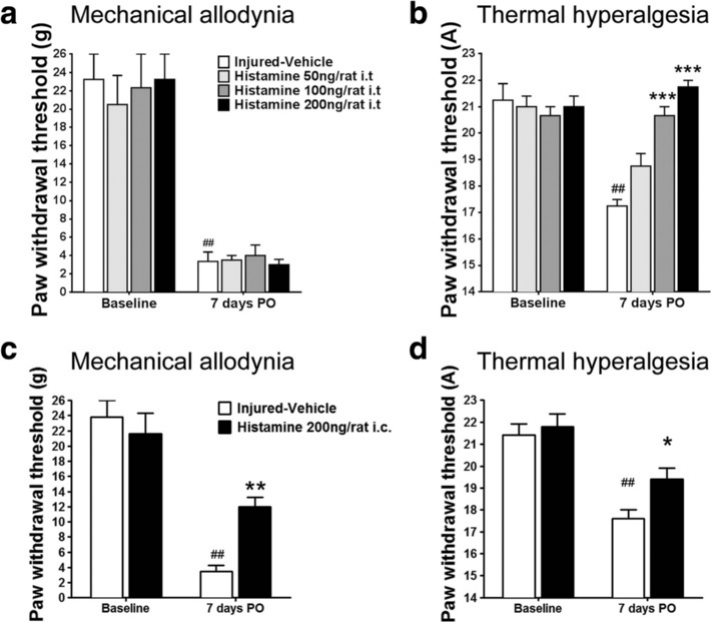
Effects of intrathecal and intracisternal histamine on thresholds to mechanical and thermal stimuli in rats. Histamine injected intrathecally once daily during days 0–7 PO at doses of 100 and 200 ng reversed heat hyperalgesia following PSL on day 7 PO but did not affect mechanical allodynia (**a**, **b**), while intracisternal histamine alleviated both mechanical and thermal hypersensitivity (**c**, **d**). *P < 0.05, **P < 0.01, and ***P < 0.001, compared with the vehicle-treated group. ^##^ *P* < 0.01, compared with the baseline. *n* = 7–9/group

HPLC assay revealed that in naive animals, histamine content in the medulla was undetectable (below the minimum detectable concentration 10 ng/g) and that in the lumbar spinal cord was 20 ± 28 ng/g. After 3 days of histidine treatment (100 mg/kg), the contents increased to 120 ± 69 ng/g in the medulla and 165 ± 90 ng/g in the lumbar spinal cord (Table 1). Moreover, histamine injected intrathecally once daily during days 0–7 PO at doses of 100 and 200 ng reversed heat hyperalgesia on day 7 PO but did not affect mechanical allodynia (Fig. 2a, b), while intracisternal histamine alleviated both mechanical and thermal hypersensitivity (Fig. 2c, d).

**Table 1:** Histamine concentrations in the medulla and lumbar cord of rats following intraperitoneal injection of histidine (100 mg/kg) once daily for 3 days

Groups	Medulla (ng/g)	Lumbar cord (ng/g)
Saline	Not detectable	20 ± 28
Histidine 100 mg/kg i.p.	120 ± 69**	165 ± 90*

*P < 0.05 and **P < 0.01 (the concentration in the medulla of saline-treated group was designated 10 ng/g, the minimum detectable concentration for our HPLC method, arbitrarily for comparison between the groups)

### Early initiation of histidine treatment was crucial for its analgesic effect

**Fig. 3:**
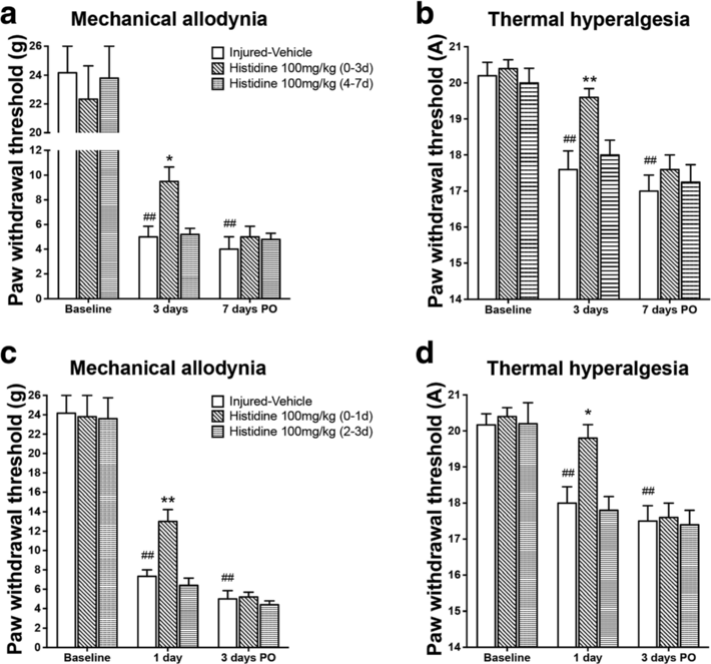
Early administration of histidine is crucial for its analgesic effect. Histidine given intraperitoneally once daily through 0–3 days PO or 0–1 day PO alleviated mechanical allodynia (**a**, **c**) and thermal hyperalgesia (**b**, **d**) following PSL during the period of treatment, whereas it had no effect if administered through 4–7 days PO or 2–3 days PO. *P < 0.05, **P < 0.01, compared with the vehicle-treated group. ^##^ *P* < 0.01, compared with the baseline. *n* = 7–9/group

We further characterized the analgesic effect of histamine. It was found that, once histidine treatment through 0–3 days PO was ceased, the difference in thresholds to mechanical and thermal stimuli between the saline- and histidine-treated groups disappeared on day 7 PO, indicating the absence of outlasting effect (Fig. 3a, b). Moreover, histidine administered from day 4 till day 7 PO (4–7 days), when the hypersensitivity had been stably established, was not able to reduce either mechanical allodynia (Fig. 3a) or thermal hyperalgesia (Fig. 3b). Furthermore, histidine treatment from the day of surgery to day 1 PO (0–1 day) (Fig. 3c and d) or to day 7 PO (0–7 days) (Additional file 1: Figure S1) significantly alleviated both mechanical allodynia and thermal hyperalgesia without outlasting effect. However, histidine treatment started from day 2 to day 3 PO (2–3 days) (Fig. 3c, d) or from day 8 to day 14 PO (8–14 days) (Additional file 1: Figure S1) did not alleviate the hypersensitivity.

### Early administration of histidine inhibited nerve injury-induced spinal microglial activation

**Fig. 4:**
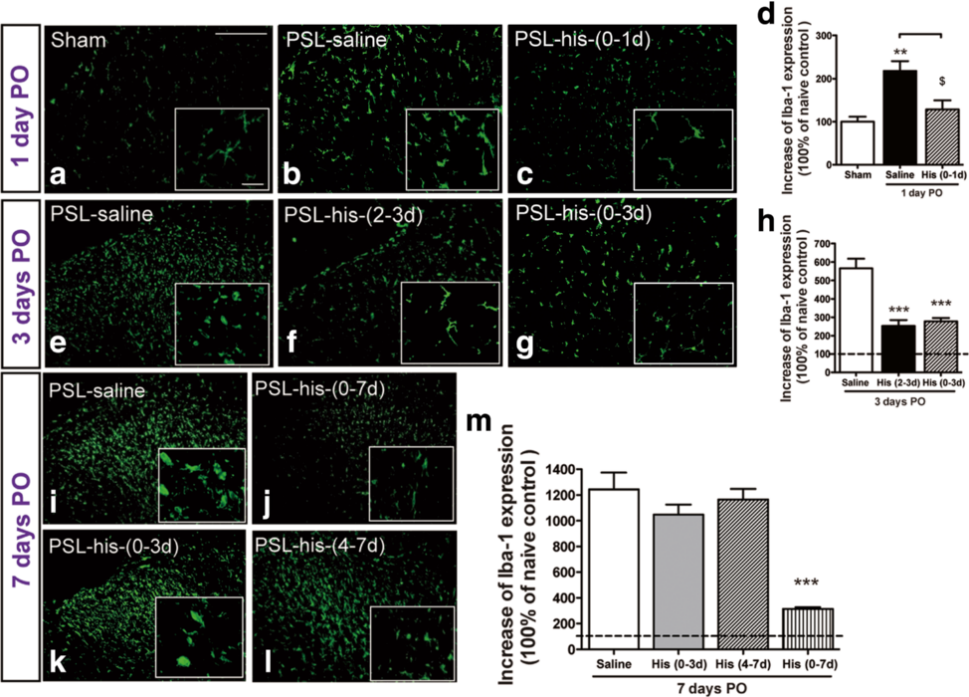
Effects of histidine on PSL-induced activation of spinal microglia. Photomicrographs show Iba-1 labeling in the lumbar spinal cord on day 1 (**a–c**), day 3 (**e–g**), and day 7 PO (**i–l**). *Insets* are high-magnification images to demonstrate multidimensional changes of spinal microglia in the ipsilateral spinal dorsal horn. **d**, **h**, and **m** represent the quantitative analysis of the area occupied by Iba-1-positive cells on day 1, day 3, and day 7 PO, respectively. Histidine (100 mg/kg, i.p.) administered once daily during the period of 0–1, 2–3, 0–3, and 0–7 days PO reduced Iba-1 expression following PSL. *Scale bar* = 200 μm (*top*) and 20 μm (*insets*). **P < 0.01, ***P < 0.001, compared with the saline-treated group. ^$^ *P* < 0.05, compared with the indicated groups. *n* = 3–4/per group

It is known that early activation of spinal microglia after nerve injury plays critical roles in the development of neuropathic pain. Therefore, we examined the effect of histidine on activation of spinal microglia following PSL. There was an increase in Iba-1 immunoreactivity condensed in the ipsilateral spinal dorsal horn beginning from day 1 PO and remaining at least till day 7 PO (Fig. 4b, e, i), indicating the long-lasting activation of spinal microglia after nerve injury. This activation was suppressed by histidine administered during the period of 0–1, 0–3, or 0–7 days PO (Fig. 4c, g, j). Administration of histidine from day 2 to day 3 PO (2–3 days) inhibited microglial activation (Fig. 4f), while administration from day 4 to day 7 PO (4–7 days) did not show such effect (Fig. 4l). Microglial activation was still observed on day 7 PO if histidine administration was stopped on day 3 PO, indicating the absence of an outlasting inhibitory effect (Fig. 4k). In addition, intrathecal histamine during the early phase inhibited spinal microglial activation in a similar way to systemic loading of histidine (Additional file 2: Figure S2).

### Inhibition of HDC enzyme or knockout of HDC gene abolished the effects of histidine on pain behavior and microglial activation

**Fig. 5:**
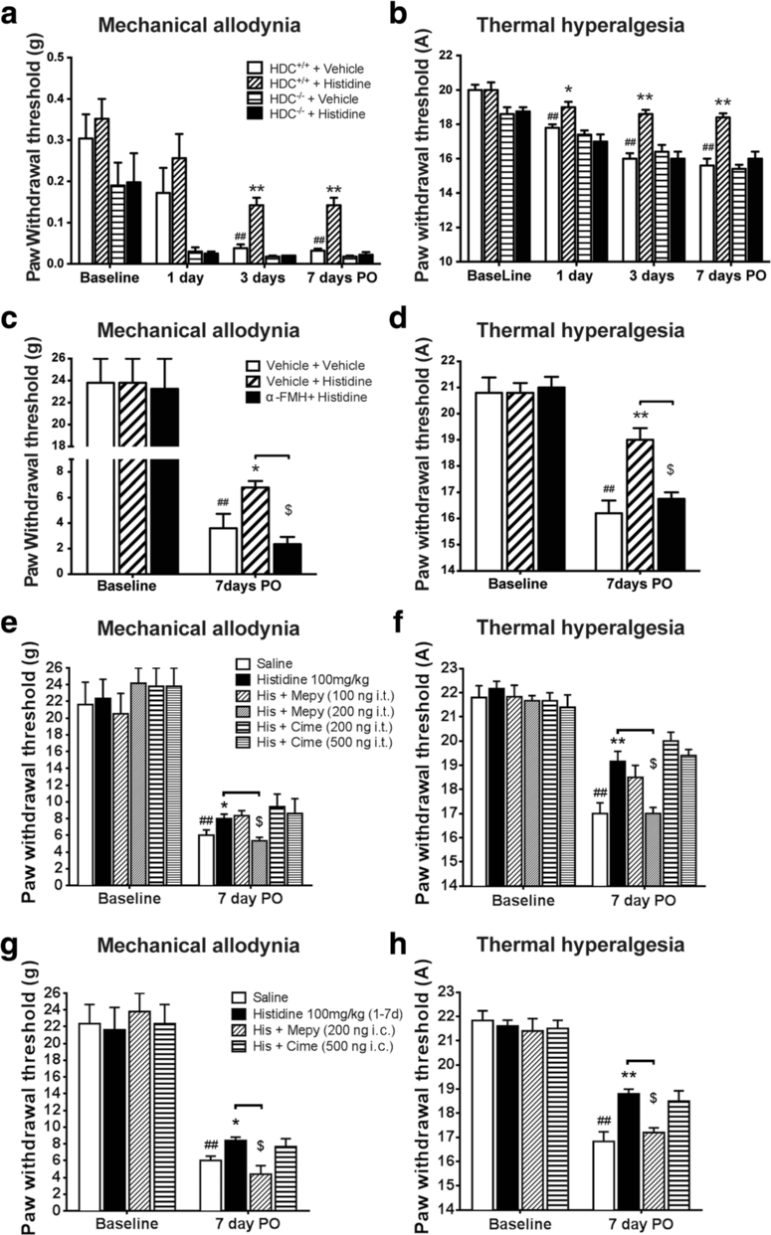
Blocking histamine synthesis or histamine H_1_ receptors abolished the analgesic effects of histidine. Histidine (200 mg/kg, i.p.) administered once daily through 0–7 days PO alleviated hypersensitivity in wild-type mice, but not in HDC^−/−^ mice (**a**, **b**). Histidine (100 mg/kg, i.p.) did not show any analgesic effect if α-FMH (50 μg, i.c.v) was administered simultaneously (**c**, **d**). Mepyramine (200 ng/rat, i.t. or i.c.) but not cimetidine (200, 500 ng/rat, i.t. or i.c.) abolished the analgesic effect of histidine (**e**–**h**). *P < 0.05, **P < 0.01, compared with the vehicle-treated group. ^##^ *P* < 0.01, compared with the baseline. ^$^ *P* < 0.05, compared with the indicated groups. *n* = 7–9/group

**Fig. 6:**
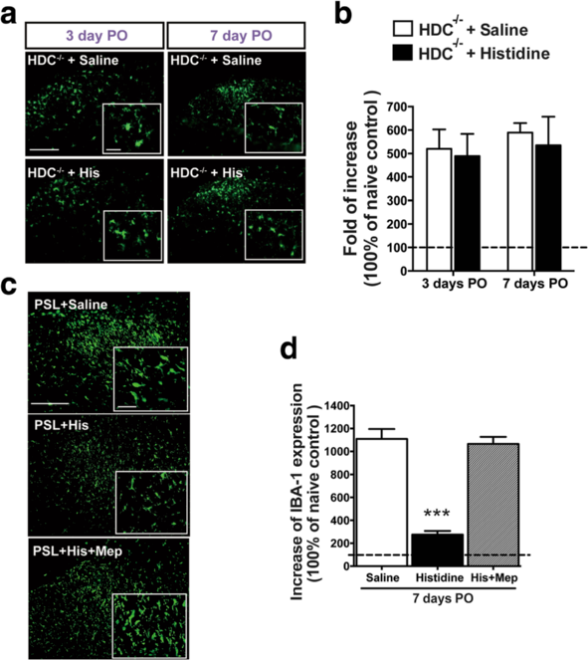
HDC gene knockout or H_1_ receptor antagonist mepyramine abolished the inhibitory effect of histidine on spinal microglial activation. Histidine (200 mg/kg, i.p.) administered once daily through 0–7 days PO did not inhibit microglial activation in HDC^−/−^ mice (**a**, **b**). Mepyramine (200 ng/rat, i.t.) abolished the inhibitory effect of histidine on microglial activation (**c**, **d**). *Scale bar* = 200 μm (*bottom*) and 20 μm (*insets*). ***P < 0.001, compared with the vehicle-treated group

Since histidine cannot be transformed to histamine without HDC enzyme, we introduced the inhibitor of HDC α-FMH and HDC gene knockout mice (HDC^−/−^) to further verify the effects of histidine are attributed to histamine. Although HDC gene deletion did not affect the development of either mechanical or heat hypersensitivity, the analgesic effect (Fig. 5a, b) and inhibitory effect on spinal microglial activation of histidine were completely abrogated (Fig. 6a, b). In addition, consecutive treatment with α-FMH (i.c.v.) for 7 days in the rats resulted in an abolishment of the analgesic effect of histidine (Fig. 5c, d).

### Histamine H_1_ receptor antagonist mepyramine abolished the analgesic effect and inhibitory effect on microglial activation of histidine

To clarify the types of histamine receptors involved in the analgesic effect of histamine, we injected mepyramine (an H_1_ receptor antagonist) or cimetidine (an H_2_ receptor antagonist) intrathecally or intracisternally 2 h after daily histidine administration during 0–7 days PO. It was found that histidine followed by mepyramine (200 ng, i.t. or intracisternally (i.c.)) was no longer able to reduce mechanical allodynia and thermal hyperalgesia (Fig. 5e–h). Meanwhile, the inhibitory effect of histidine on microglial activation in the dorsal horn of the lumbar cord was also abolished by intrathecal mepyramine (Fig. 6c, d). By contrast, cimetidine (200, 500 ng, i.t. or 500 ng, i.c.) did not influence the analgesic effect of systemic histidine (Fig. 5e–h).

### Histidine reduced IL-1β upregulation in the spinal cord induced by partial sciatic nerve injury

**Fig. 7:**
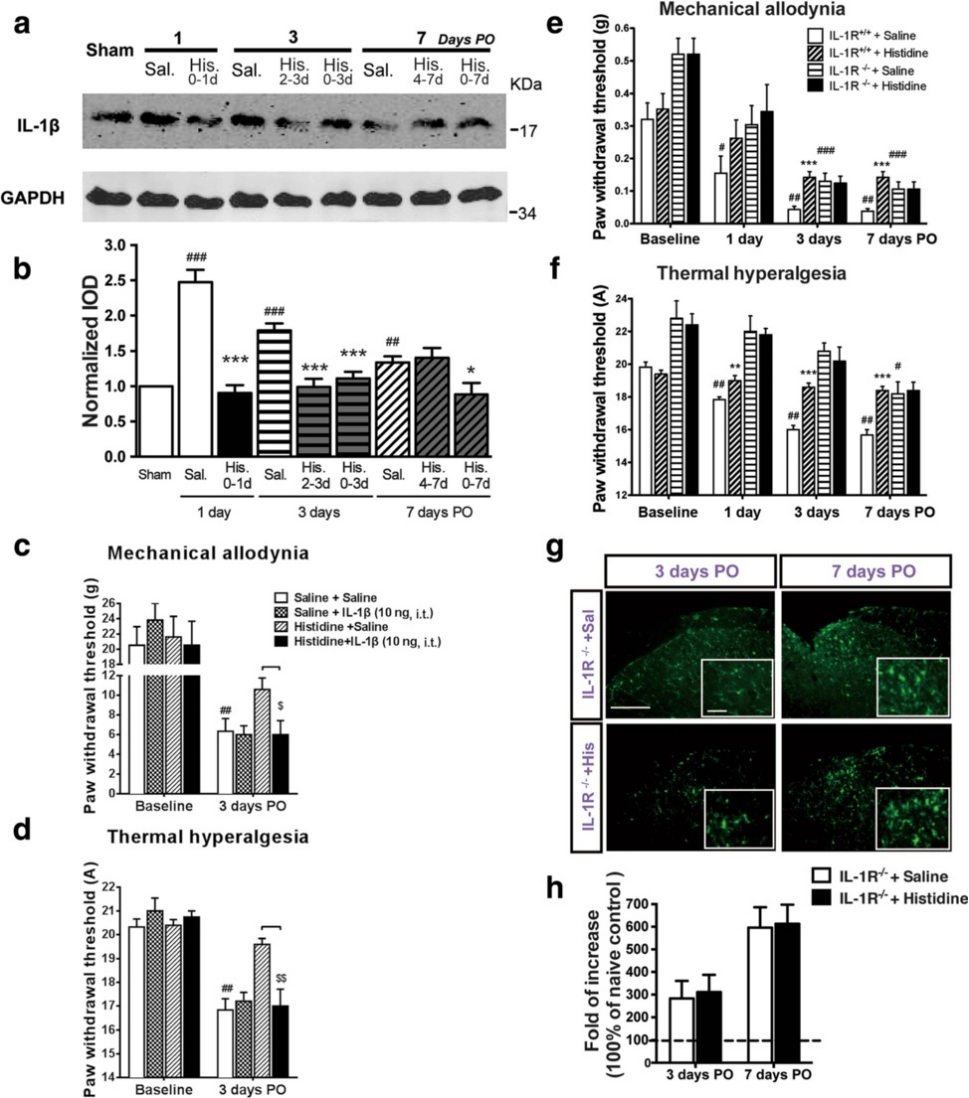
Histidine reduced IL-1β upregulation in the spinal cord induced by PSL. Histidine (100 mg/kg, i.p.) administered once daily during the period of 0–1, 2–3, 0–3, and 0–7, but not 4–7 days PO, reduced the increase in levels of IL-1β (**a**, **b**). Histidine (100 mg/kg, i.p.) did not show any analgesic effect if IL-1β (10 ng, i.t.) was administered 2 h later (**c**, **d**) in 3 days PO. Histidine (200 mg/kg, i.p.) administered through 0–7 days PO did not affect hypersensitivity and microglial activation following PSL in IL-1R^−/−^ mice (**e**–**h**). *Scale bar* = 200 μm (*bottom*) and 20 μm (*insets*). *P < 0.05, **P < 0.01, and *** *P* < 0.001 compared with the indicated groups. ^#^ *P* < 0.05, ^##^ *P* < 0.01, and ^###^ *P* < 0.001, compared with the baseline. ^$^ *P* < 0.05 and ^$$^ *P* < 0.01, compared with the indicated groups. *n* = 7–9/group

The levels of proinflammatory cytokine IL-1β (the active form) at different time points (1, 3, and 7 days PO) were measured to further explore whether the inhibitory effect of histidine on microglial activation is associated with the downregulation of microglial function. IL-1β production increased abruptly and reached its peak within 1 day after nerve injury and maintained at higher levels than the sham group through 7 days PO. Histidine treatment suppressed the production of IL-1β in a pattern parallel to that on microglia activation. That is, histidine initiated from the day of surgery, regardless for 1, 3, or 7 days or administered during 2–3 days PO, inhibited IL-1β production, but administration during 4–7 days PO did not affect IL-1β production (Fig. 7a, b).

To verify the involvement of IL-1β inhibition in the analgesic effect of histidine, we injected IL-1β intrathecally 2 h after daily histidine administration for 3 days and found that histidine was no longer able to alleviate mechanical allodynia and thermal hyperalgesia (Fig. 7c, d). Moreover, although the IL-1R^−/−^ mice also developed hypersensitivity to mechanical and thermal stimuli in 7 days after PSL, the severity was lower and the onset was delayed compared with the wild-type mice (Fig. 7e, f). The inhibitory effects of histamine on behavior and spinal microglial activation were not observed in the IL-1R^−/−^ mice (Fig. 7e–h).

## Discussion

Previously, we found that central histamine is able to suppress autotomy behavior following peripheral neurectomy in rats, demonstrating the analgesic effect of central histamine on the development of phantom pain [15]. In the present study, we found histidine (0–1, 0–3, 0–7, and 0–14 days, PO) significantly alleviated both thermal hyperalgesia and mechanical allodynia following partial sciatic nerve ligation. Moreover, compared with mechanical allodynia, thermal hyperalgesia appeared more responsive to histidine, since it was alleviated by a lower dose of histidine and in a dose-dependent manner (Fig. 1). Based on the knowledge that systemic histidine increases the cerebral levels of histamine [8, 9], together with the following evidence obtained from the present study, (1) systemic administration of histidine increased histamine levels in the medulla and lumbar spinal cord; (2) intrathecal and intracisternal injection of histamine prevented the development of neuropathic pain; (3) and inhibition of HDC enzyme or HDC gene knockout, both of which interrupt the synthesis of histamine from histidine, abolished the analgesic effect of histidine; and (4) both intrathecal and intracisternal injection of H_1_ receptor antagonist mepyramine antagonized the analgesic effect of histidine, we conclude that the analgesic effect of histidine is largely attributed to central histamine.

It is reported that intracerebroventricular pretreatment with zolantidine (a histamine H_2_ receptor antagonist), but not mepyramine, abrogated the anti-hypersensitivity effect of central histamine in the spinal nerve ligation (SNL) model of neuropathic pain [27]. The analgesic effect of central histamine on acute trigeminal pain can be abolished by intracerebroventricular ranitidine (a histamine H_2_ receptor antagonist) pretreatment, but not by chlorpheniramine (a histamine H_1_ receptor antagonist) pretreatment [13]. However, our results suggest that central histamine, via H_1_ receptors both in spinal and medullar levels, is able to alleviate neuropathic pain of CRPS-2 induced by PSL. This conflict implies that analgesic effect of central histamine may involve different receptor subtypes depending on the sites in the CNS, although these studies consistently demonstrated the analgesic effect of central histamine. Another possible explanation may be that the pathophysiological mechanisms are different between PSL-induced neuropathic pain and others.

We are interested to find that although histidine reduced pain hypersensitivity if given throughout postoperative days, it did not show analgesic effect if given from day 2, 4 (Fig. 3), or 8 PO (Additional file 1: Figure S1). These results not only indicate that early initiation of histamine is crucial for its analgesic effect on neuropathic pain in the PSL model of CRPS-2 but imply that central endogenous histamine may act as an analgesic factor in pain modulation as well. It has been noted that pain or sensory abnormalities predominates in acute/early stage of CRPS [28], and the early and late phases of neuropathic pain following nerve injury share different pathophysiology and pain networks [29, 30]. Thus, this critical time window for analgesic effect suggests that histamine may act on some crucial event(s) that occur in the early phase of PSL-induced neuropathic pain. Intriguingly, however, we found that behavioral hypersensitivity relapsed if histidine administration was ceased either on day 2, 4 (Fig. 3), or 8 PO (Additional file 1: Figure S1). The absence of an outlasting effect indicates the crucial event(s) that histamine acts on may take place early but tonically be active at least for 14 days after PSL injury and plays key roles in the initiation and maintenance of neuropathic pain in CRPS-2.

Spinal microglial activation occurs during the early phase of neuropathic pain and has been linked to central sensitization and initiation of neuropathic pain [31–33]. We found microglial activation in the lumber spinal cord occurred within 1 day following PSL and lasted for at least 7 days (Fig. 4), demonstrating the early activation of spinal microglia. Interestingly, similar to the effect on behavioral hypersensitivity, histidine treatment initiated immediately (0–1, 0–3, and 0–7 days, PO), but not 3 days after PSL, inhibited microglial activation. In addition to demonstrate the critical role of spinal microglial activation in the development of neuropathic pain in CRPS-2, these results also provide evidence for the early phase-specific analgesic effect of central histamine from the aspect of inhibition of microglial activation. The findings that histidine concurrently failed to inhibit microglial activation and behavioral hypersensitivity in HDC^−/−^ mice (Fig. 6a, b) as well as in mepyramine-treated rats (Fig. 6c, d) also support that the analgesic effect of central histamine may relate to the inhibition of spinal microglia.

Activation of microglia is indicated not only by changes in morphology but also by the increase in the production and secretion of various cytokines and chemokines, including IL-1β, IL-6, and tumor necrosis factor-a (TNF-α) [34]. In this study, we found that IL-1β, the functional index of microglial activation, was upregulated during 7 days PO with a peak at 24 h after PSL. It is very likely that the prompt IL-1β release in response to nerve injury contributes to neuropathic pain in the PSL model, as does in other models [30, 35, 36]. Moreover, histidine flattened the peak of IL-1β production on day 1 PO and significantly inhibited IL-1β expression if administered in a regimen that was able to inhibit behavioral hypersensitivity and microglial activation (Fig. 7a, b), but lost its effect on hypersensitivity or microglial activation in the IL-1β-treated rats (Fig. 7c, d) and IL-1R^−/−^ mice (Fig. 7e, h) which express comparable levels of H_1_ receptors with their wild-type littermates in the lumbar spinal cord (data not shown). These results together support the early critical time window for the analgesic effect of central histamine and the involvement of IL-1β inhibition.

Intriguingly, we also found that inhibition of microglial activation later than the first day PO does not guarantee an attenuation of hypersensitivity. That is, the time window for interrupting microglial activation to prevent neuropathic pain induced by PSL might be much more limited than previously identified in other models of neuropathic pain [30, 32]. Therefore, we propose that, in order to prevent the development of neuropathic pain in CRPS-2, intervention aiming to inhibit the activation of microglia should be initiated immediately after nerve injury. Moreover, our results suggest approaches that can increase the release of central histamine, such as histamine H_3_ receptor antagonists, may be options to relieve neuropathic pain of CRPS-2 in the very early phase but may not benefit patients with established CRPS-2, although an acute robust increase of central histamine has been reported to ameliorate allodynia following PSL [14]. On the other hand, the first-generation H_1_ receptor antagonists that have permeability to the CNS should be avoided in patients with this disease because they potentially neutralize the analgesic effect of central histamine.

In addition, it has been reported that an early inhibition of microglial activation can attenuate hypersensitivity following nerve injury [37, 38]. To our surprise, however, the present study found that although histidine administered on the second and third days PO (2-3 days, PO) suppressed microglial activation as well as IL-1β production, it had no effect on pain hypersensitivity (Fig. 4f, h). These findings are supportive to the studies that reported the disassociation between microglial inhibition (indicated by microglial markers such as Iba1) and analgesic outcomes [39–41]. This finding also implies that the hypersensitivity later than 1 day after PSL may not solely depend on microglial activation. Moreover, since histidine administered during days 4–7 PO did not inhibit microglial activation (Fig. 4l, m), we speculate that histamine presumably acts on some early event(s), which occurs soon after PSL injury and triggers the activation of microglia, but not directly on microglia. This speculation is partially supported by the recent finding that only a very little percentage (less than 10 %) of microglia from the brain responds to histamine [42].

Unlike systemic histidine, intrathecal injection of histamine abolished thermal hyperalgesia, but had no effect on mechanical allodynia following PSL. Since histamine levels are increased all over the CNS by systemic histidine, but increased only locally in lower segments of the spinal cord by intrathecal histamine, it seems that histamine receptors in supraspinal structures may be involved in the anti-allodynic effect of histidine. This was supported by the finding that intracisternal injection of histamine alleviated both mechanical allodynia and thermal hyperalgesia (Fig. 2c and d). Moreover, intracisternal injection of H_1_ receptor antagonist mepyramine, rather than H_2_ receptor antagonist cimetidine, antagonized the analgesic effect of systemic histidine (Fig. 5g, h). These results may imply that pathophysiological changes in the spinal level following partial sciatic nerve injury are more associated with thermal hyperalgesia, while those in supraspinal level are associated with mechanical allodynia in addition to thermal hyperalgesia. This speculation is partially supported by the previous studies reporting that ascending input to nucleus gracilis is critical to the manifestation of tactile allodynia [43–45]. Therefore, it is possible that histamine may inhibit both mechanical allodynia and thermal hyperalgesia of CRPS-2 through acting on H_1_ receptors at the supraspinal levels.

## Conclusions

The present study found that central histamine via spinal and supraspinal H_1_ receptors alleviates neuropathic pain in the early phase of CRPS-2 induced by partial nerve injury in rodents. The coincident inhibition of spinal microglial activation and IL-1β upregulation early after nerve injury may contribute to this effect. These results suggest that intervention approaches should be initiated as early as possible for the treatment of neuropathic pain component of CRPS-2, while CNS-penetrating H_1_ receptor antagonists should be avoided.

## Abbreviations

α-FMH, α-fluoromethylhistidine; BCA, bicinchoninic acid; CNS, central nervous system; CRPS-2, complex regional pain syndrome type 2; GAPDH, glyceraldehyde-3-phosphate dehydrogenase; HDC, histidine decarboxylase; IL-1R, IL-1 receptor; PO, postoperatively; PSL, partial sciatic nerve ligation

## Additional files

Additional file 1: Figure S1. Effects of histidine administered in other regimens on mechanical allodynia (A) and thermal hyperalgesia (B) following PSL in rats. Histidine (100 mg/kg, i.p.) or saline was administered once daily during the period of 0–14 days PO, 0–7 days PO, or 8–14 days PO. **P* < 0.05, ***P* < 0.01, and ****P* < 0.001, compared with the saline-treated group. ^#^ *P* < 0.05, ^##^ *P* < 0.01, compared with the baseline. *n* = 7–9/group. (TIF 1,201 kb) Additional file 2: Figure S2. Effect of intrathecal histamine (HA) on PSL-induced activation of spinal microglia. Histamine (200 ng/rat, i.t.) on the first day PO (0–1 day) (B) or 2–3 days PO (2–3 days) (E) reduced Iba-1 expression following PSL. C and F represent the quantitative analysis of the area occupied by Iba-1-positive cells on day 1 and day 3 PO, respectively. Scale bar = 200 μm (top) and 20 μm (insets). ***P* < 0.01, compared with the saline-treated group. *n* = 3–4/per group. (TIF 2,148 kb)

## References

[1] Borchers AT, Gershwin ME (2013). Complex regional pain syndrome: a comprehensive and critical review. Autoimmun Rev.

[2] International Association for the Study of Pain Subcommitteeon Taxonomy (1986). Classification of chronic pain. Descriptions of chronic pain syndromes and definitions of pain terms. Pain Suppl.

[3] Stanton-Hicks MD, Burton AW, Bruehl SP, Carr DB, Harden RN, Hassenbusch SJ (2002). An updated interdisciplinary clinical pathway for CRPS: report of an expert panel. Pain Pract.

[4] de Mos M, Huygen FJ, van der Hoeven-Borgman M, Dieleman JP, Ch Stricker BH, Sturkenboom MC (2009). Outcome of the complex regional pain syndrome. Clin J Pain.

[5] Christensen K, Jensen EM, Noer I (1982). The reflex dystrophy syndrome response to treatment with systemic corticosteroids. Acta Chir Scand.

[6] Marinus J, Moseley GL, Birklein F, Baron R, Maihofner C, Kingery WS (2011). Clinical features and pathophysiology of complex regional pain syndrome. Lancet Neurol.

[7] Haas HL, Sergeeva OA, Selbach O (2008). Histamine in the nervous system. Physiol Rev.

[8] Lozeva V, Tarhanen J, Attila M, Mannisto PT, Tuomisto L (2003). Brain histamine and histamine H3 receptors following repeated L-histidine administration in rats. Life Sci.

[9] Yoshimatsu H, Chiba S, Tajima D, Akehi Y, Sakata T (2002). Histidine suppresses food intake through its conversion into neuronal histamine. Exp Biol Med (Maywood).

[10] Kamei C, Ishizawa K, Kakinoki H, Fukunaga M (1998). Histaminergic mechanisms in amygdaloid-kindled seizures in rats. Epilepsy Res.

[11] Tamaddonfard E, Rahimi S (2004). Central effect of histamine and peripheral effect of histidine on the formalin-induced pain response in mice. Clin Exp Pharmacol Physiol.

[12] Malmberg-Aiello P, Lamberti C, Ghelardini C, Giotti A, Bartolini A (1994). Role of histamine in rodent antinociception. Br J Pharmacol.

[13] Tamaddonfard E, Khalilzadeh E, Hamzeh-Gooshchi N, Seiednejhad-Yamchi S (2008). Central effect of histamine in a rat model of acute trigeminal pain. Pharmacol Rep.

[14] Huang L, Adachi N, Nagaro T, Liu K, Arai T (2007). Histaminergic involvement in neuropathic pain produced by partial ligation of the sciatic nerve in rats. Reg Anesth Pain Med.

[15] Yu J, Lou GD, Yue JX, Tang YY, Hou WW, Shou WT (2013). Effects of histamine on spontaneous neuropathic pain induced by peripheral axotomy. Neurosci Bull.

[16] Bruehl S (2010). An update on the pathophysiology of complex regional pain syndrome. Anesthesiology.

[17] Chopra P, Cooper MS (2013). Treatment of complex regional pain syndrome (CRPS) using low dose naltrexone (LDN). J Neuroimmune Pharmacol.

[18] Li WW, Guo TZ, Shi X, Czirr E, Stan T, Sahbaie P (2014). Autoimmunity contributes to nociceptive sensitization in a mouse model of complex regional pain syndrome. Pain.

[19] Tajerian M, Sahbaie P, Sun Y, Leu D, Yang HY, Li W (2015). Sex differences in a murine model of complex regional pain syndrome. Neurobiol Learn Mem.

[20] Seltzer Z, Dubner R, Shir Y (1990). A novel behavioral model of neuropathic pain disorders produced in rats by partial sciatic nerve injury. Pain.

[21] Shir Y, Seltzer Z (1990). A-fibers mediate mechanical hyperesthesia and allodynia and C-fibers mediate thermal hyperalgesia in a new model of causalgiform pain disorders in rats. Neurosci Lett.

[22] Shir Y, Seltzer Z (1991). Effects of sympathectomy in a model of causalgiform pain produced by partial sciatic nerve injury in rats. Pain.

[23] Wang F, Cai B, Li KC, Hu XY, Lu YJ, Wang Q (2015). FXYD2, a gamma subunit of Na(+), K(+)-ATPase, maintains persistent mechanical allodynia induced by inflammation. Cell Res.

[24] Zhang SH, Yu J, Lou GD, Tang YY, Wang RR, Hou WW (2016). Widespread pain sensitization following partial infraorbital nerve transection in MRL/MPJ mice. Pain.

[25] Fan YY, Hu WW, Dai HB, Zhang JX, Zhang LY, He P (2011). Activation of the central histaminergic system is involved in hypoxia-induced stroke tolerance in adult mice. J Cereb Blood Flow Metab.

[26] Light M, Minor KH, De Witt P, Jasper KH, Davies SJ (2012). Multiplex array proteomics detects increased MMP-8 in CSF after spinal cord injury. J Neuroinflammation.

[27] Wei H, Jin CY, Viisanen H, You HJ, Pertovaara A (2014). Histamine in the locus coeruleus promotes descending noradrenergic inhibition of neuropathic hypersensitivity. Pharmacol Res.

[28] Bruehl S, Harden RN, Galer BS, Saltz S, Backonja M, Stanton-Hicks M (2002). Complex regional pain syndrome: are there distinct subtypes and sequential stages of the syndrome?. Pain.

[29] Inoue M, Rashid MH, Fujita R, Contos JJ, Chun J, Ueda H (2004). Initiation of neuropathic pain requires lysophosphatidic acid receptor signaling. Nat Med.

[30] Kawasaki Y, Xu ZZ, Wang X, Park JY, Zhuang ZY, Tan PH (2008). Distinct roles of matrix metalloproteases in the early- and late-phase development of neuropathic pain. Nat Med.

[31] Gosselin RD, Suter MR, Ji RR, Decosterd I (2010). Glial cells and chronic pain. Neuroscientist.

[32] Ji RR, Suter MR (2007). p38 MAPK, microglial signaling, and neuropathic pain. Mol Pain.

[33] Zhuang ZY, Wen YR, Zhang DR, Borsello T, Bonny C, Strichartz GR (2006). A peptide c-Jun N-terminal kinase (JNK) inhibitor blocks mechanical allodynia after spinal nerve ligation: respective roles of JNK activation in primary sensory neurons and spinal astrocytes for neuropathic pain development and maintenance. J Neurosci.

[34] Zhuo M, Wu G, Wu LJ (2011). Neuronal and microglial mechanisms of neuropathic pain. Mol Brain.

[35] Lindenlaub T, Sommer C (2003). Cytokines in sural nerve biopsies from inflammatory and non-inflammatory neuropathies. Acta Neuropathol.

[36] Maixner DW, Yan X, Gao M, Yadav R, Weng HR (2015). Adenosine monophosphate-activated protein kinase regulates interleukin-1beta expression and glial glutamate transporter function in rodents with neuropathic pain. Anesthesiology.

[37] Ledeboer A, Sloane EM, Milligan ED, Frank MG, Mahony JH, Maier SF (2005). Minocycline attenuates mechanical allodynia and proinflammatory cytokine expression in rat models of pain facilitation. Pain.

[38] Raghavendra V, Tanga F, DeLeo JA (2003). Inhibition of microglial activation attenuates the development but not existing hypersensitivity in a rat model of neuropathy. J Pharmacol Exp Ther.

[39] Tsuda M, Shigemoto-Mogami Y, Koizumi S, Mizokoshi A, Kohsaka S, Salter MW (2003). P2X4 receptors induced in spinal microglia gate tactile allodynia after nerve injury. Nature.

[40] Leinders M, Knaepen L, De Kock M, Sommer C, Hermans E, Deumens R (2013). Up-regulation of spinal microglial Iba-1 expression persists after resolution of neuropathic pain hypersensitivity. Neurosci Lett.

[41] Gallo A, Dimiziani A, Damblon J, Michot B, Des Rieux A, De Kock M (2015). Modulation of spinal glial reactivity by intrathecal PPF is not sufficient to inhibit mechanical allodynia induced by nerve crush. Neurosci Res.

[42] Pannell M, Szulzewsky F, Matyash V, Wolf SA, Kettenmann H (2014). The subpopulation of microglia sensitive to neurotransmitters/neurohormones is modulated by stimulation with LPS, interferon-gamma, and IL-4. Glia.

[43] Kim HY, Wang J, Gwak YS (2012). Gracile neurons contribute to the maintenance of neuropathic pain in peripheral and central neuropathic models. J Neurotrauma.

[44] Ossipov MH, Zhang ET, Carvajal C, Gardell L, Quirion R, Dumont Y (2002). Selective mediation of nerve injury-induced tactile hypersensitivity by neuropeptide Y. J Neurosci.

[45] Sun H, Ren K, Zhong CM, Ossipov MH, Malan TP, Lai J (2001). Nerve injury-induced tactile allodynia is mediated via ascending spinal dorsal column projections. Pain.

