# Supplementary figures and images for: A critical time window for the analgesic effect of central histamine in the partial sciatic ligation model of neuropathic pain
Source: J Neuroinflammation. 2016 Jun 24;13:163. doi: 10.1186/s12974-016-0637-0 (PMC4921020; doi:10.1186/s12974-016-0637-0)

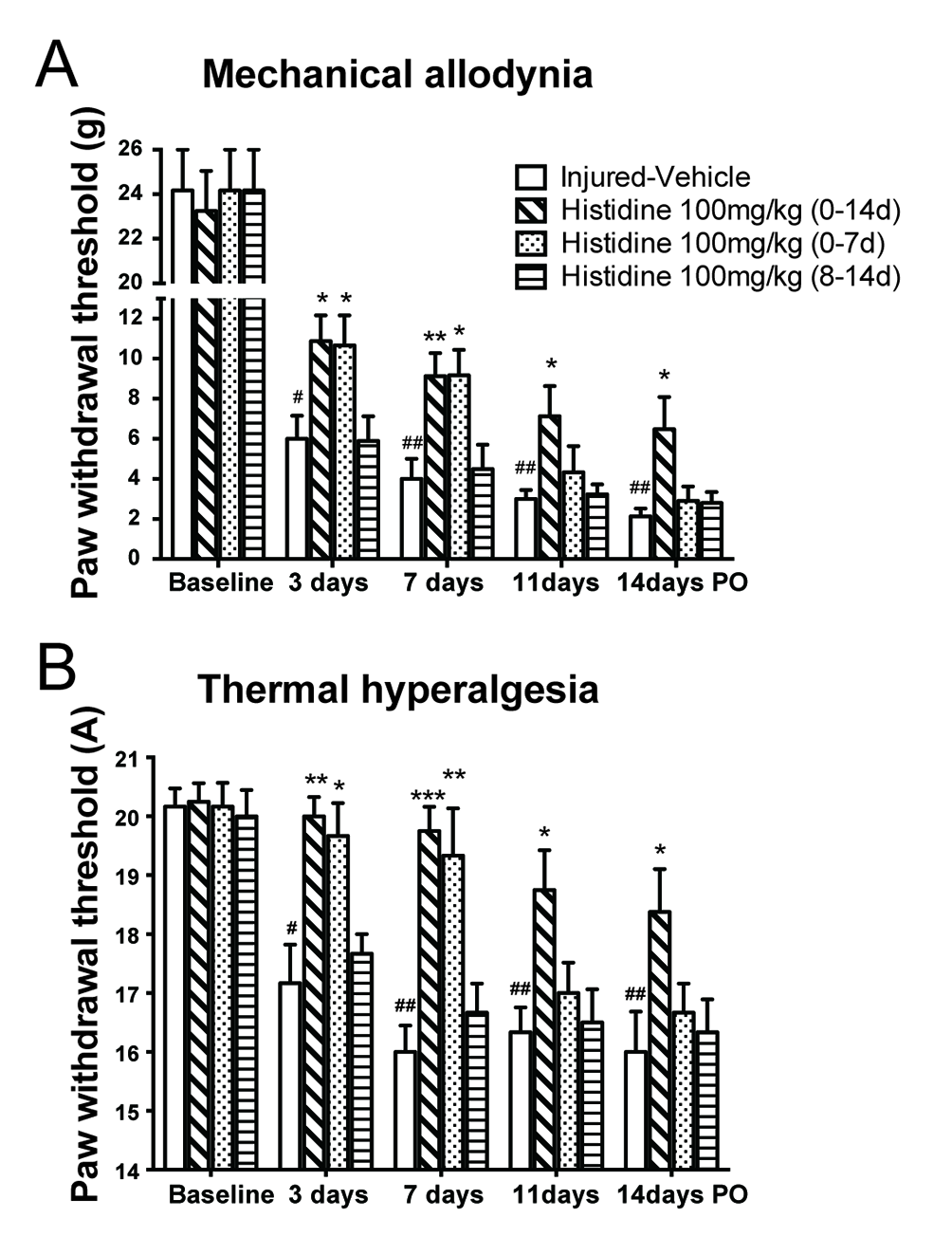

Supplement: Additional file 1: Figure S1. — Effects of histidine administered in other regimens on mechanical allodynia (A) and thermal hyperalgesia (B) following PSL in rats. Histidine (100 mg/kg, i.p.) or saline was administered once daily during the period of 0–14 days PO, 0–7 days PO, or 8–14 days PO. *P < 0.05, **P < 0.01, and ***P < 0.001, compared with the saline-treated group. # P < 0.05, ## P < 0.01, compared with the baseline. n = 7–9/group. (TIF 1,201 kb) [file 12974_2016_637_MOESM1_ESM.tif]

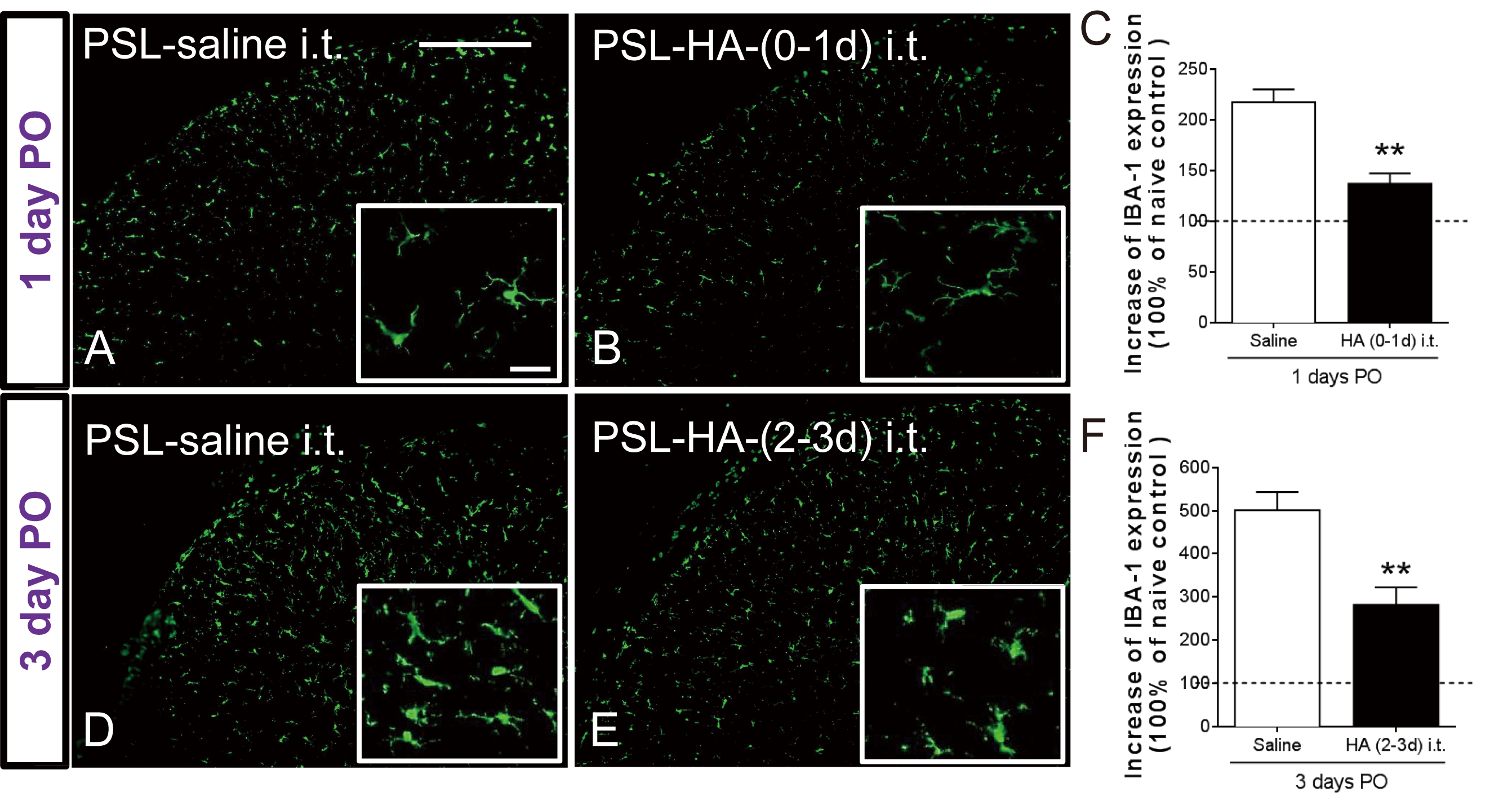

Supplement: Additional file 2: Figure S2. — Effect of intrathecal histamine (HA) on PSL-induced activation of spinal microglia. Histamine (200 ng/rat, i.t.) on the first day PO (0–1 day) (B) or 2–3 days PO (2–3 days) (E) reduced Iba-1 expression following PSL. C and F represent the quantitative analysis of the area occupied by Iba-1-positive cells on day 1 and day 3 PO, respectively. Scale bar = 200 μm (top) and 20 μm (insets). **P < 0.01, compared with the saline-treated group. n = 3–4/per group. (TIF 2,148 kb) [file 12974_2016_637_MOESM2_ESM.tif]
